# From Reactive to Predictive One Health: AI-Enabled Frameworks for Integrated Zoonotic Surveillance and Governance

**DOI:** 10.3390/ijerph23070850

**Published:** 2026-06-29

**Authors:** Elena Sorrentino, Alessandra Mazzeo, Celestina Mascolo, Michele Valentino Chiara, Sebastiano Rosati, Lucia Maiuro

**Affiliations:** 1 Department of Agricultural, Environmental and Food Sciences (DiAAA), University of Molise, Via Francesco de Sanctis snc, 86100 Campobasso, Italy; sorrentino@unimol.it (E.S.); alessandramazzeo@unimol.it (A.M.); 2 Department of Prevention, Complex Structure Animal Health, Local Health Agency of Caserta, Via Feudo San Martino, 81100 Caserta, Italy; celeste.mascolo@gmail.com; 3 Unit of Prevention and Veterinary Public Health, General Directorate for Health Protection and Coordination of the Regional Health System, Centro Direzionale C3, 80132 Naples, Italy; 4 Sector of Collective Prevention and Public and Veterinary Health Sector, General Directorate for Health Protection and Coordination of the Regional Health System, Centro Direzionale C3, 80132 Naples, Italy; michelevalentino.chiara@regione.campania.it; 5 Department of Agricultural, Forestry and Food Sciences (DISAFA), University of Turin, Largo Paolo Braccini 2, 10095 Grugliasco, Italy

**Keywords:** One Health, artificial intelligence, zoonoses, data integration, epidemiological surveillance, decision-support systems, public health, food safety, disease prevention, predictive modeling

## Abstract

**Highlights:**

**Public health relevance—How does this work relate to a public health issue?**

- Structural and digital silos hinder the operationalization of One Health.
- The *Salmonella* Umbilo outbreak highlights restricted cross-sectoral data access.

**Public health significance—Why is this work of significance to public health?**

- Federated Learning and FAIR principles may support privacy-preserving AI surveillance.
- Multi-matrix data integration supports predictive zoonotic risk modeling.

**Public health implications—What are the key implications or messages for practitioners, policy makers and/or researchers in public health?**

- AI-enabled surveillance may help reduce disparities in Inner Areas and low-resource settings.
- The OH-IS framework aligns with the EU AI Act and the European Health Data Space.

**Abstract:**

The operationalization of the One Health (OH) approach remains a major challenge due to persistent fragmentation across human, animal, and environmental data systems. This gap is exacerbated by climate change, which acts as a risk multiplier for pathogen transmission and agri-food system vulnerability. Drawing on more than a decade of research, including the re-emergence of brucellosis in Italy and the 2024 *Salmonella* Umbilo outbreak, this perspective discusses key weaknesses in current data management, particularly the lack of real-time, interoperable data sharing. To address these challenges, we propose an AI-enabled One Health Information System (OH-IS), grounded in FAIR data principles and privacy-preserving architectures. The proposed conceptual framework integrates multi-matrix data streams, combining Earth observation data, genomic surveillance through whole-genome sequencing (WGS), and livestock mobility within a geospatially integrated architecture to support timely decision-making in vulnerable settings. By analyzing the constraints of siloed databases, we discuss how automated semantic harmonization could conceptually support improved risk assessment and outbreak reconstruction in recent zoonotic events. This approach may facilitate a transition from descriptive to anticipatory surveillance, providing a scalable model to move One Health from a conceptual paradigm toward a more integrated and data-driven surveillance framework aligned with EU digital health policies and global health security priorities.

## 1. Introduction

The One Health (OH) approach has emerged as a comprehensive framework for addressing the complex interconnections between human, animal, and environmental health, particularly in the context of zoonotic and foodborne diseases [1,2]. Recent studies emphasize the importance of capturing socioecological interconnections within the OH framework, pointing to the need for approaches that simultaneously address human, animal, and environmental health dimensions rather than treating them as separate domains [3,4,5,6,7,8,9]. The need for the OH approach is further reinforced by the increasing frequency of zoonotic disease occurrence, the spread of antimicrobial resistance (AMR), and ongoing global environmental changes [10]. In this interconnected context, where environmental processes, agricultural ecosystems, food systems, and human populations are closely linked, the OH approach represents not only a conceptual paradigm but also an operational necessity [11].

Zoonotic diseases represent a substantial proportion of infectious diseases affecting humans, with many emerging pathogens originating from animal reservoirs [2,6,12,13]. In the European Union (EU), key foodborne zoonotic agents, including *Campylobacter* spp., *Salmonella* spp., and *Listeria monocytogenes*, remain major drivers of cross-border outbreaks and are associated with severe clinical outcomes [14]. These pathogens circulate across livestock populations, environmental matrices, and the food supply chain, highlighting food microbiology as a critical interface within the OH framework [15,16,17].

Despite its strong conceptual foundation, the operationalization of the OH approach remains limited. One of the major limitations lies in the persistent fragmentation of data across human, veterinary, environmental, and food-related sectors [18,19]. Surveillance systems often operate in parallel rather than in an integrated manner, resulting in siloed databases that hinder interoperability, delay information exchange, and limit the ability to reconstruct epidemiological events in a timely manner. These limitations are further exacerbated by the lack of real-time data sharing across sectors, which constrains the timely integration of epidemiological, genomic, and environmental information.

Drawing on over a decade of field investigations and outbreak analyses, this perspective highlights key weaknesses in current data governance. Evidence from recent zoonotic events exemplifies these challenges. The multi-country *Salmonella* Umbilo outbreak in 2024 [20] revealed critical limitations in cross-sectoral data accessibility. While genomic data enabled the identification of a common source of infection in the food chain, the lack of interoperable access across human and veterinary data systems hindered the integration of cross-sectoral evidence, constraining the full reconstruction of transmission pathways [4,8,21]. These constraints underscore the need for standardized data-sharing frameworks and interoperable infrastructures to support integrated OH investigations. In particular, the development of real-time data integration frameworks is essential to bridge the gap between local surveillance and applied genomic epidemiology [7,22]. Similarly, long-term surveillance of brucellosis in southern Italy has shown that the disease persists in high-density livestock areas. This pattern is influenced by the interactions between farm management practices and environmental factors. In particular, local hydrographic networks may act as potential environmental reservoirs, contributing to pathogen persistence and spread [23]. These dynamics are likely to be further exacerbated by climate-driven changes, which reshape interactions across the human–animal–environment interface [23,24,25].

In addition to these epidemiological constraints, economic and structural barriers further limit the implementation of effective OH strategies [13]. Furthermore, optional control programs for non-mandatory diseases are often underused due to economic difficulties and limited financial capacity at both the farm and territorial levels, particularly in vulnerable territories such as “Inner Areas”, defined as geographically peripheral areas characterized by limited access to essential services, infrastructures, and innovation opportunities [26]. The application of standardized control strategies across heterogeneous territories often overlooks local environmental, socio-economic, and infrastructural differences. As a result, “one-size-fits-all” approaches tend to be less effective in disease control programs, particularly in high-risk and resource-limited settings where tailored, context-specific interventions are required [26,27,28,29]. Taken together, these structural and financial limitations underscore the need for integrated, data-driven solutions capable of maximizing existing resources.

In recent years, digital technologies have emerged as powerful tools with increasing relevance for both current and future OH applications. Artificial intelligence (AI) and machine learning offer the potential to integrate heterogeneous datasets, enable real-time signal detection, and generate predictive risk maps, thereby overcoming the latency and fragmentation of traditional surveillance systems [29,30,31,32]. When combined with interoperable Open Data infrastructures and FAIR (Findable, Accessible, Interoperable, Reusable) principles [33], which have been successfully adapted to multi-stakeholder health data ecosystems [34,35], AI can transform fragmented information into actionable knowledge across the human–animal–environment interface. In this context, Federated Learning (FL) offers a robust solution for multi-institutional collaboration, enabling privacy-preserving data integration while maintaining local data control across sectors [36]. Recent advances further highlight the importance of explainable AI (XAI) to ensure transparency, interpretability, and stakeholder trust in cross-sectoral decision-making and governance processes within OH systems [37,38,39,40].

In this perspective, climate change should be considered a cross-cutting risk multiplier within the OH framework. By altering environmental conditions, host–pathogen interactions, and ecosystem stability, climate dynamics contribute to the emergence, persistence, and transmission of zoonotic and foodborne hazards [10,37]. At the same time, emerging environmental reservoirs such as the “Plastisphere” (microbial communities associated with microplastics), where microplastics act as ecological niches that support pathogen persistence and dissemination, as well as drivers of AMR [41], further complicate traditional surveillance. AI-driven environmental mapping and risk modeling are essential for capturing these dynamic interfaces, particularly as microplastic-associated biofilms are increasingly recognized as hotspots for resistance gene exchange, including in agricultural environments where microplastics and their associated plastisphere, are widely distributed in both surface waters and soils [41,42,43,44,45].

Within the European regulatory landscape, the transition toward interoperable data systems is explicitly supported by key policy frameworks, including the EU Animal Health Law [46] and the Farm to Fork Strategy [47]. Beyond regional policy, this approach resonates with the United Nations 2030 Agenda for Sustainable Development [48], particularly SDG 3 (Good Health and Well-being), SDG 13 (Climate Action), and SDG 15 (Life on Land). Collectively, these policy and development frameworks provide a robust foundation for deploying AI-enabled surveillance systems that are both territorially adaptive and globally aligned.

However, implementation remains uneven, particularly in environmentally and socio-economically vulnerable areas. To prevent a widening digital divide in public health, the proposed framework emphasizes scalable, lightweight AI solutions and federated data architectures that support capacity building even in resource-limited settings [49]. In this context, recent assessments of European mountain and Inner Areas suggest that achieving these targets requires value-chain approaches capable of integrating environmental stewardship, socio-economic resilience, and cross-sectoral data governance [27,28].

While several OH surveillance frameworks and integrated monitoring initiatives have been proposed [18,19,50,51], most remain constrained by limited interoperability, insufficient consideration of environmental reservoirs, and the absence of scalable mechanisms for real-time cross-sectoral data exchange. Moreover, available examples of integrated systems are often focused on specific hazards or implemented at national and international scales, where reduced timeliness and granularity may limit response at the local level [7]. Consequently, important gaps remain in the operational integration of environmental reservoirs, cross-sectoral data interoperability, and economically sustainable implementation pathways. The proposed One Health Information System (OH-IS) framework seeks to address these challenges, focusing not on developing novel technologies, but on their conceptual integration within a unified One Health perspective through: (i) the explicit integration of hydrographic and plastisphere dynamics as surveillance matrices; (ii) Federated Learning and FAIR principles as core architectural components; (iii) predictive surveillance linked to the “In-Embryo” financial model; and (iv) alignment with emerging European regulatory frameworks, including the EU AI Act and the EHDS [52]. Together, these elements provide a conceptual blueprint for a more integrated, scalable, and territorially adaptive OH surveillance architecture.

This perspective was developed through the synthesis of evidence derived from field investigations, outbreak analyses, policy and regulatory documents, and peer-reviewed literature addressing One Health surveillance, data interoperability, environmental risk factors, and AI-enabled systems. Despite its interdisciplinary scope, the framework is organized around a single core message: interoperable and privacy-preserving data architectures may support more integrated, territorially adaptive, and anticipatory One Health governance. The selected examples were included to illustrate recurring operational barriers and opportunities for integration across the human–animal–environment interface rather than to provide a systematic review of the available evidence.

Accordingly, this article proposes a conceptual and policy-oriented OH-IS framework intended to guide future implementation, testing, and validation efforts. Empirical assessment through pilot implementations, comparative modeling, or performance benchmarking is beyond the scope of the present contribution and represents an important direction for future research.

To address these interconnected gaps, AI-enabled OH-IS may provide a valuable pathway toward overcoming structural barriers to OH implementation [4,13,23,49,53,54,55]. Within this perspective, an integrated architecture combining genomic, spatial, and environmental data is outlined to support anticipatory, evidence-based decision-making within the OH paradigm. This approach is consistent with emerging European policy frameworks such as the EU AI Act and the European Health Data Space (EHDS) [52]. Ultimately, this digital transformation could contribute to a more resilient and predictive OH security architecture [56].

## 2. Structural and Digital Barriers in Current One Health Surveillance

### 2.1. The Silo Effect and the Challenge of Real-Time Data Sharing

**Figure 1:**
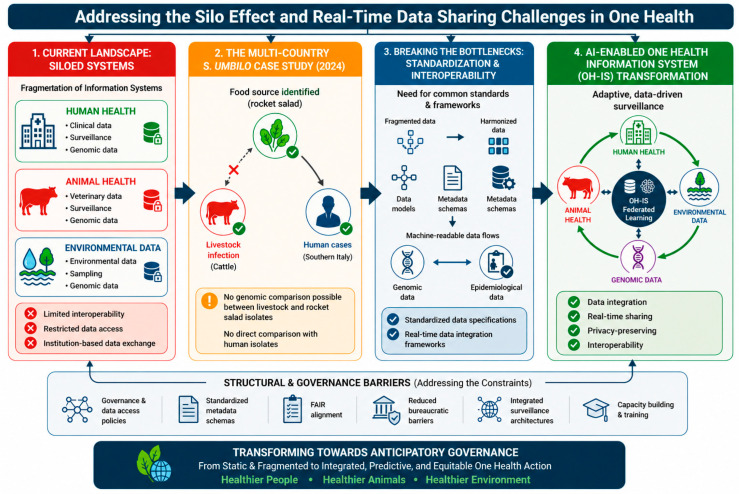
From siloed data systems to integrated OH surveillance: conceptual framework of structural barriers and AI-enabled solutions. The figure highlights current fragmentation across human, animal, and environmental domains, illustrates limitations observed during the 2024 *Salmonella* Umbilo outbreak, and outlines the transition toward interoperable, real-time, and AI-driven OH-IS.

The fragmentation of information systems remains a major bottleneck for the operationalization of the OH approach. Currently, human, veterinary, and environmental data are managed through independent architectures that rarely achieve true interoperability. This siloed structure prevents a holistic understanding of zoonotic events, as data exchange often relies on formal institutional requests rather than automated, standardized, and machine-readable information flows. Addressing these bottlenecks requires standardized data specifications to maximize the utility of pathogens [57], alongside integrated frameworks specifically designed for real-time surveillance and applied genomic epidemiology [7]. These structural bottlenecks and their implications for OH surveillance are summarized in Figure 1.

The 2024 multi-country *Salmonella* Umbilo outbreak represents a clear example of these structural limitations. Despite the widespread adoption of Whole-Genome Sequencing (WGS) in national reference laboratories, the restricted accessibility and cross-sectoral integration of genomic data from pathogen isolates, together with associated epidemiological metadata, continue to constrain integrative analyses. This confirms that technological advancements alone remain insufficient without full semantic and technical interoperability across sectors [18]. This gap is increasingly addressed through privacy-preserving architectures, such as FL, which enable multi-institutional collaboration without compromising data sovereignty or security [36]. Recent studies further emphasize that, although WGS provides high-resolution genomic data for pathogen identification and source tracing, its effectiveness is constrained by persistent challenges related to data standardization, interoperability, and infrastructure integration [58].

Similar challenges have been reported across a range of One Health surveillance initiatives. A systematic review of One Health surveillance systems identified recurrent barriers including fragmented data management, insufficient cross-sectoral data sharing, lack of harmonized data standards, and legal constraints related to data ownership and confidentiality [51]. More recently, analyses of the MERS outbreak response highlighted persistent barriers to timely data sharing, including fragmented responsibilities across sectors, insufficient One Health collaboration, and the lack of common mechanisms for sharing outbreak-related data [59]. These findings indicate that challenges related to interoperability, governance, and cross-sectoral coordination remain unresolved despite advances in surveillance technologies. Recent analyses of OH data integration frameworks further indicate that even where integrated genomic surveillance platforms exist, effective One Health implementation requires not only data integration but also joint interpretation, investigation, and response across human, animal, and environmental sectors [7].

These limitations reflect not only technical constraints but also governance-related barriers, including bureaucratic clearance procedures and the absence of protocols aligned with FAIR principles for cross-sectoral data integration. In addition, the restricted accessibility of publicly generated genomic and epidemiological data, often confined to institutional or sector-specific use, further delays outbreak reconstruction and scientific interpretation [4,18]. As observed in our previous investigations, restricted access to raw genomic and spatial datasets during early outbreak phases reduces the predictive capacity of public health systems, shifting responses from proactive prevention to reactive containment [57,60,61]. These structural barriers align with recognized gaps in cross-sectoral coordination, as highlighted by the OH Surveillance Codex, which emphasizes that without standardized data interoperability and shared governance, surveillance systems remain sectorial and fragmented [50]. Systematic assessments of OH surveillance architectures further confirm that most current systems lack integrated data flows, standardized metadata, and real-time feedback mechanisms, limiting their capacity to support anticipatory governance [51].

Traditional approaches based on centralized databases, manual data harmonization, or static reporting protocols have proven insufficient to address the complexity of cross-sectoral surveillance [62]. These models rely on delayed, fragmented, and often non-standardized data flows, limiting their capacity to support real-time decision-making. In contrast, AI-enabled systems allow dynamic semantic harmonization and continuous integration of heterogeneous data streams, representing a paradigm shift from static interoperability to adaptive, data-driven surveillance. Until shared data governance and standardized metadata schemas are established, the capacity to mitigate the health and economic impacts of foodborne zoonoses will remain structurally constrained, underscoring the urgent need for interoperable, privacy-preserving, and AI-ready surveillance architectures.

### 2.2. Environmental Reservoirs and Territorial Vulnerability: The Case of Brucellosis

The persistence and re-emergence of zoonotic diseases in specific geographic areas highlight the limitations of surveillance systems that focus primarily on the animal host while neglecting environmental compartments [63,64,65]. While re-emergence has recently been reported in European countries previously considered free of bovine brucellosis [65], a well-documented example of long-term persistence is represented by southern Italy, where the disease remains endemic in areas characterized by high livestock density and complex environmental interactions [60,63,64,66]. This evidence aligns with a growing body of literature demonstrating that effective control of zoonotic diseases requires integrated preventive strategies that extend beyond host-targeted interventions, incorporating environmental monitoring, advanced diagnostics, and early warning systems within an OH framework [13,26,67].

Despite decades of mandatory eradication programs, the failure to achieve disease eradication in these areas suggests that current surveillance models do not adequately account for the role of the environment as a dynamic reservoir [64,66]. Previous research has suggested that local hydrographic networks and water systems may act as conduits for pathogen persistence and dispersal, although the current evidence remains largely observational and warrants further investigation across different epidemiological settings [23,68,69]. These local dynamics are increasingly exacerbated by climate-driven shifts that reshape zoonotic risk at the human–animal–environment interface [24], highlighting a significant knowledge gap in current OH frameworks regarding the water–environment interface [70]. The lack of integration between veterinary surveillance data and environmental monitoring, such as water quality, flow dynamics, and soil contamination, creates a critical blind spot in risk assessment, limiting the identification of non-animal and wildlife-related infection sources. Territorial vulnerability further exacerbates these challenges. These limitations are particularly evident in Inner Areas, where geographical isolation, reduced infrastructure, and limited access to advanced monitoring systems amplify the fragmentation of surveillance. In such contexts, the absence of real-time data acquisition and integrated information systems significantly delays outbreak detection and response, reinforcing structural vulnerabilities within the OH framework. These territorial challenges align with broader analyses of European mountain regions and Inner Areas, which emphasize that sustainable development requires integrated value chains capable of linking local production, environmental stewardship, and socio-economic resilience [27,28]. In fact, the application of standardized control strategies across heterogeneous territories often fails to account for local environmental, socio-economic, and infrastructural differences. The efficacy of disease prevention programs may be diminished by this “one-size-fits-all” strategy, especially in high-risk and resource-constrained settings where tailored, context-specific interventions are required [26,28].

Traditional extensive farming practices, including seasonal transhumance and free-range grazing in mountainous Inner Areas, pose a significant challenge to static surveillance models, as animal movements and environmental exposures are highly dynamic and often unrecorded. The lack of real-time spatial tracking in these contexts creates blind spots that delay early warning signals and hinder cross-sectoral coordination. Recent data suggest that animal movements in extensive systems are far more complex than previously assumed. GPS-based monitoring, for instance, has uncovered frequent intermingling between herds from different holdings, as well as interactions with wildlife and movements beyond designated grazing boundaries. These previously unrecorded contact networks reveal a hidden layer of transmission risk that traditional models fail to capture [60,71,72]. These diverse challenges share a common denominator: static surveillance systems may not fully capture dynamic, cross-compartment transmission pathways. This spatiotemporal heterogeneity, driven by environmental reservoirs, animal mobility, territorial vulnerability, and wildlife-mediated transmission pathways, represents a fundamental limitation of existing approaches, reinforcing the need for integrated, adaptive systems capable of modeling complex interactions across the human–animal–environment interface [40].

Without a transition toward integrated, spatially explicit, and data-driven surveillance architectures, current approaches remain largely reactive and territorially disconnected. These limitations underscore a fundamental requirement for the proposed OH-IS: the need to move beyond host-centric surveillance toward a multi-compartment approach that integrates environmental, epidemiological, and territorial data. Effective spatiotemporal modeling conceptually requires consistent data granularity, standardized ontologies, and interoperable geospatial layers—conditions that legacy systems currently fail to meet.

## 3. The Integrated OH-IS Framework: A Multi-Matrix AI Approach

To overcome the structural barriers identified in current surveillance, we propose a conceptual framework for an AI-enabled OH-IS. This system is designed not merely as a centralized database, but as a dynamic, interoperable ecosystem capable of harmonizing heterogeneous data streams for monitoring and detection to support anticipatory and evidence-based governance [62]. In this perspective, integrating climatic, environmental, and territorial data, particularly through GIS-based analysis, is essential, as it enables the identification of both intervention urgency and priority areas for action. By linking epidemiological signals with spatial and environmental drivers, as well as agricultural systems, livestock production, and food chain dynamics, the OH-IS can support more timely, risk-based, and spatially targeted decision-making.

**Figure 2:**
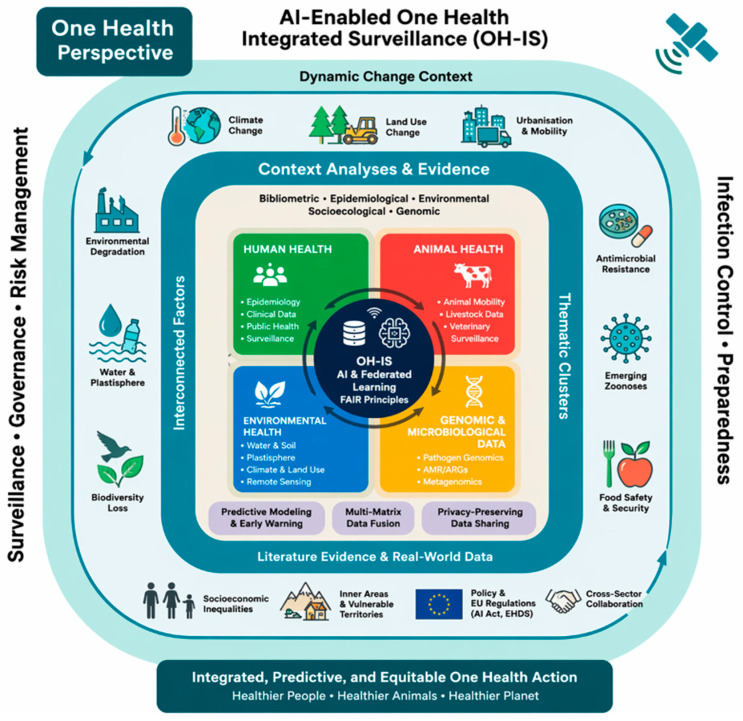
Conceptual framework of the OH approach within a dynamic socioecological context. The figure illustrates the interconnected domains of human, animal, environmental, and genomic systems, influenced by drivers such as climate change, land use, and urbanization. These interactions define the complexity of zoonotic disease dynamics and provide the contextual basis for the development of AI-enabled, integrated OH-IS.

A conceptual overview of the OH system and its socioecological drivers is presented in Figure 2, providing the contextual framework for the proposed AI-enabled OH-IS architecture.

To preserve conceptual focus, the OH-IS is presented as a modular conceptual architecture rather than as a fully operational platform. Interoperable data integration, privacy-preserving analytics, semantic harmonization, and explainable decision support represent its core components, while climate, hydrographic, genomic, environmental, and economic dimensions are discussed as application-specific layers.

### 3.1. Technical Architecture: Federated Learning and FAIR Data Streams

The core of the proposed conceptual OH-IS architecture is grounded in FL, a decentralized machine learning paradigm in which models are trained across distributed nodes, such as regional veterinary laboratories, hospital information systems, and environmental monitoring agencies, without the need to share raw sensitive data. While FL represents an established paradigm in generic healthcare informatics, this framework conceptualizes its systematic translation into the One Health domain to address critical challenges related to data privacy, regulatory fragmentation, and limited cross-sector interoperability. By enabling distributed data processing while preserving local data ownership, FL reduces barriers associated with data access constraints and governance heterogeneity, allowing the integration of sensitive datasets without requiring centralized storage, which is often associated with legal and security risks. This decentralized paradigm is increasingly recognized as a cornerstone for ethical medical AI, providing a systematic solution for privacy-preserving and equitable data ecosystems in complex healthcare environments [36].

To ensure that these decentralized data streams could be effectively translated into actionable insights, the proposed OH-IS would implement automated semantic harmonization and FAIR principles [33]. By leveraging standardized ontologies and interoperable metadata schemas, the system dynamically aligns WGS-derived pathogen clusters, GIS spatial layers, and epidemiological reports across sectors and data domains [18].

This architecture directly addresses the fragmentation and rule misalignment identified in current surveillance systems, enabling standardized, machine-readable data exchange and improving comparability across sectors and jurisdictions. Recent studies further highlight that effective FAIR implementation in complex, multi-source systems depends on structured metadata models, community standards, and semantic interoperability, particularly in environmental and health-related data ecosystems [73,74,75,76]. These approaches transform fragmented datasets into an integrated knowledge framework, enabling the identification of non-obvious correlations between distal environmental drivers, such as hydrographic flow dynamics or microplastic-associated biofilms, and proximal infection events.

**Figure 3:**
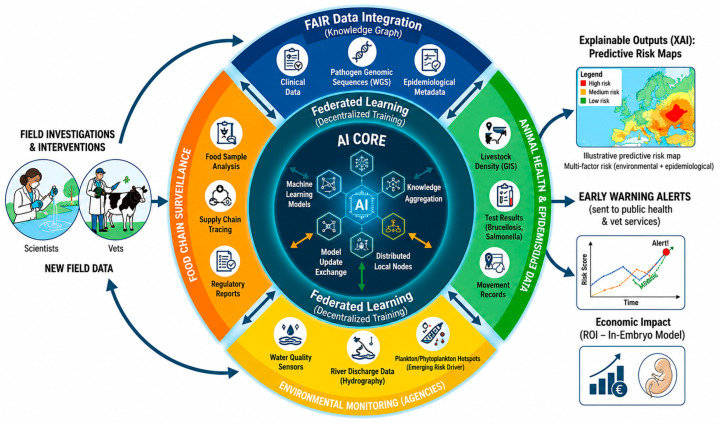
Operational architecture of the AI-enabled One Health Information System (OH-IS). The framework is designed to integrate multi-sectoral data streams (human, animal, food, and environmental) through Federated Learning (FL) and FAIR principles, enabling privacy-preserving data integration and semantic harmonization into a unified Knowledge Graph. The AI core generates explainable outputs (XAI), including predictive risk maps, early warning alerts, and decision-support tools, supporting iterative feedback and anticipatory, data-driven OH governance.

The integrated operational architecture of the proposed AI-enabled OH-IS is illustrated in Figure 3, highlighting the interaction between decentralized data sources, semantic harmonization processes, and AI-driven outputs. The Knowledge Graph serves as the semantic backbone of the OH-IS, enabling dynamic linkage among heterogeneous entities, including pathogen clusters, geographic coordinates, temporal metadata, environmental variables, and epidemiological records. By leveraging standardized ontologies and interoperable metadata structures, it supports semantic harmonization, cross-domain querying, and AI-assisted inference. This integration layer facilitates the interpretability of relationships across the human–animal–environment interface and provides the semantic foundation for explainable AI-driven outputs. As an illustrative scenario, local WGS metadata, georeferenced water-quality indicators, and anonymized epidemiological signals could be conceptually linked through the Knowledge Graph, with technical implementation requiring co-design with domain experts and institutional stakeholders.

In addition to data integration, the proposed OH-IS would incorporate explainable AI (XAI) components to ensure transparency, interpretability, and trust in decision-making processes.

To bridge the gap between digital infrastructure and field-level implementation, the proposed OH-IS is designed to integrate multiple data domains, including climatic and environmental data, remote sensing, livestock monitoring systems, and georeferenced field records, to support continuous validation and system recalibration. These heterogeneous data streams enable dynamic risk assessment and real-time, evidence-based decision-making [7,57].

Within the proposed architecture, Federated Learning, FAIR-based semantic harmonization, the Knowledge Graph, and Explainable AI represent the core components required to ensure interoperability, privacy-preserving analytics, and transparent decision support. Additional modules, including plastisphere risk mapping, specific Precision Livestock Farming integrations, advanced remote-sensing applications, or regional climate downscaling, may be implemented according to territorial priorities, data availability, and resource constraints. This modular design supports scalability while allowing context-specific adaptation across diverse OH settings.

Beyond traditional laboratory and clinical reporting, the integration of high-frequency and multi-source data enhances the temporal resolution of surveillance and enables early detection of anomalies. This approach supports multi-level early warning systems, including predictive risk maps, alert thresholds, and inspection prioritization, facilitating the translation of surveillance signals into actionable decisions across OH sectors [77].

### 3.2. Data Sources and Field-Level Integration in One Health Surveillance

To fully operationalize the OH-IS framework, the integration of high-resolution, multi-source data streams at the field level is essential. Beyond traditional laboratory and clinical reporting, the proposed system would incorporate heterogeneous data sources that capture the dynamic interactions across agricultural systems, livestock production, and environmental interfaces.

In this context, Precision Livestock Farming (PLF) technologies represent a key component of data acquisition. Remote animal monitoring systems, based on wearable sensors and IoT devices, enable the continuous tracking of biometric parameters, behavioral patterns, and environmental exposure, generating real-time, high-frequency datasets at the farm level [62,78,79].

Complementing on-farm monitoring, geospatial tracking technologies—including GPS-based systems, geofencing algorithms, and remote sensing tools—enable the characterization of animal movements and interactions across landscapes. AI-driven anomaly detection can identify deviations from expected mobility patterns, supporting the detection of high-risk contacts and potential breaches of movement restrictions, particularly in extensive and transhumant production systems where traditional surveillance is inherently limited [71,72].

The integration of these data streams is particularly relevant in territorially vulnerable contexts such as Inner Areas, where environmental exposure, livestock density, and infrastructural constraints intersect. In these settings, limited access to advanced monitoring systems and fragmented surveillance infrastructures delay outbreak detection and response. The deployment of distributed, sensor-based monitoring systems within the OH-IS framework provides a scalable solution to overcome these limitations, enabling continuous and context-sensitive surveillance [26,60].

In high-density livestock systems, such as water buffalo (*Bubalus bubalis*) production in southern Italy, the interaction between intensive farming practices and complex hydrographic networks amplifies the risk of pathogen persistence and environmental dissemination. Environmental contamination pathways, including water flow dynamics and waste management systems, play a critical role in shaping transmission patterns across the human–animal–environment interface [23]. In this context, the proposed OH-IS could integrate real-time environmental mapping and automated biosecurity monitoring to identify contamination pathways, supporting targeted interventions and improving risk mitigation in high-density production systems. These mobility datasets can then be integrated with environmental and biosecurity information to identify high-risk interactions, support compliance monitoring, and refine risk-based interventions.

The effectiveness of this multi-source integration relies on the adoption of standardized data specifications and automated data processing pipelines, which are essential to maximize the utility of heterogeneous datasets and ensure interoperability across sectors [7,57]. By combining environmental, animal, and epidemiological data streams within a unified analytical framework, the OH-IS could enhance the temporal and spatial resolution of surveillance, enabling early detection of emerging risks and supporting more timely, evidence-based decision-making.

Overall, the integration of high-frequency, field-level data within the OH-IS transforms traditional surveillance systems from static and retrospective models into dynamic, adaptive infrastructures capable of capturing complex, cross-compartment interactions in real time. This transition could be key to supporting anticipatory governance and effective risk mitigation within the OH paradigm.

### 3.3. Environmental Interfaces and Integrated Risk Modeling in One Health Surveillance

A key conceptual contribution of the proposed OH-IS is the integration of the water–plastisphere nexus as a dynamic surveillance matrix, highlighting an environmental reservoir that is often overlooked by traditional epidemiological surveillance. Traditional risk models often treat aquatic systems as static compartments; in contrast, this framework conceptualizes hydrographic networks as active and interconnected conduits for pathogen dispersal and antimicrobial resistance (AMR) gene exchange [80].

Spatiotemporal modeling of hydrographic and climatic dynamics enables the correlation between rainfall intensity, river discharge, and seasonal temperature variability, and the downstream spread of zoonotic pathogens across watersheds [23]. Climate-induced sanitation challenges, such as contamination of water sources during floods and droughts, further exacerbate the dissemination of drug-resistant pathogens and antimicrobial resistance genes (ARGs) across the human–animal–environment interface [81]. Recent advances in FAIR-based environmental data ecosystems highlight the importance of integrating high-resolution time-series data for dynamic monitoring and predictive modeling of environmental processes [82]. In parallel, plastisphere risk mapping represents an additional analytical layer within the OH-IS. AI-driven analysis applied to satellite imagery and multispectral sensors enables the identification of microplastic accumulation hotspots, which act as vectors and ecological niches that facilitate microbial persistence, biofilm formation, and horizontal gene transfer (HGT) [80,83]. Microplastic-associated biofilms within hydrographic networks may therefore function as persistent environmental reservoirs, extending pathogen survival and amplifying downstream contamination risks.

By integrating environmental monitoring, real-time animal mobility data, and epidemiological datasets, the OH-IS enables a multi-compartment, system-level representation of disease dynamics across the human–animal–environment interface. This approach supports the identification of high-risk contact zones, where hydrographic networks, environmental contamination sources, and animal movements converge, thereby enhancing the predictive performance and spatial targeting of surveillance systems. Comparable dynamics have been documented in aquaculture systems, where environmental conditions and production practices jointly shape microbial ecology and system-level sustainability [84]. A paradigmatic application of this approach is found in shellfish farming and coastal supply chains, where the integration of meteo-marine parameters, microbiological indicators, and spatial modeling enables the anticipation of contamination risks in bivalve molluscs and supports timely, risk-based management decisions. Systems such as MytilEX, which combine in situ monitoring, high-performance computing, and artificial intelligence, demonstrate how environmental data streams can be translated into decision-support tools for competent authorities and for the sustainable management of aquaculture systems [85]. In these contexts, addressing the water–animal interface through integrated monitoring is essential for advancing truly interoperable OH surveillance systems [70].

A similar integrative approach can be applied to the prediction and management of vector-borne and environmentally mediated infectious diseases, including both viral and bacterial pathogens, by combining epidemiological, entomological, veterinary, and environmental surveillance within a unified early warning system. Such systems enable optimized vector monitoring, targeted sampling strategies, and enhanced risk communication, while supporting the timely activation of local authorities for coordinated intervention measures [86].

## 4. Implementation Pathways and Policy Integration

The transition from fragmented surveillance systems to an AI-enabled OH-IS requires a strategic roadmap that addresses economic sustainability, regulatory alignment, and technological adoption. The key differences and comparative advantages of the proposed OH-IS framework, compared with traditional surveillance models, are summarized in Table 1.

### 4.1. Economic Sustainability and the “In-Embryo” Financial Model

One of the primary obstacles to implementing integrated OH systems is the perceived high cost associated with digital transformation and cross-sectoral coordination. However, growing evidence indicates that the lack of integrated surveillance generates substantially higher economic burdens, including reduced livestock productivity, trade restrictions, and reactive public health expenditures [4,13,32,60,63,87,88]. Zoonotic and animal infectious diseases negatively impact breeding efficiency and increase production costs, reducing market competitiveness and constraining the transition toward sustainable farming systems envisioned under the EU Green Deal [13,60,63,89,90,91].

To address this challenge, we propose the adoption of an “In-Embryo” Financial Model, which prioritizes proactive resource allocation during the early stages of disease emergence and system development. The “In-Embryo” Financial Model is conceptually grounded in preventive health economics [13,32]. Its underlying assumption is that early investments in integrated surveillance infrastructure may generate economic returns through avoided outbreak-related costs, including livestock losses, culling expenses, trade restrictions, public health interventions, and supply-chain disruptions [13,32,92]. In addition, earlier risk detection may contribute to preserving market access, supporting territorial resilience, and reducing long-term response expenditures. To establish a clear conceptual delimitation, it must be emphasized that this component functions at present as a qualitative blueprint rather than a formalized, algorithmic economic model.

At its current stage, the model should be regarded as a conceptual framework intended to support future economic evaluation rather than as a validated economic instrument. Consequently, its dynamic economic functions remain only partially developed within this contribution, serving as a heuristic narrative to guide future transdisciplinary research; its operational deployment remains strictly contingent upon prospective empirical calibration involving context-specific epidemiological baselines, outbreak cost estimates, and stakeholder-specific cost–benefit analyses.

By providing a framework for estimating the potential return on investment (ROI) associated with AI-driven predictive mapping, including avoided outbreaks, reduced culling, and preserved market access, this model supports more informed multi-stakeholder investment strategies. In this way, the OH-IS can be reframed from a cost center into a strategic investment, aimed at safeguarding the economic sustainability of the entire food supply chain, particularly in vulnerable Inner Areas [13,26,92].

In this perspective, AI functions not only as a diagnostic tool, but also as an enabler of predictive economic modeling and decision support. By facilitating early warning systems and anticipatory risk mapping, it supports a shift from reactive interventions, such as large-scale culling, toward preventive, risk-based biosecurity strategies, in line with the growing international recognition of AI as a key enabler of predictive surveillance and proactive investment in zoonotic risk management [56].

The territorial applicability of this model is particularly relevant in complex production contexts. In high-density livestock systems such as water buffalo farming, intensive production coupled with complex hydrographic networks can amplify pathogen dispersal and environmental contamination risks. A conceptual AI-enabled framework can integrate real-time environmental mapping and automated biosecurity monitoring to identify contamination pathways, although its operational deployment would require context-specific validation and stakeholder co-design. Similarly, traditional extensive practices such as seasonal transhumance challenge static surveillance models by introducing dynamic and spatially distributed animal movements. A conceptual OH-IS architecture can integrate spatiotemporal modeling approaches to capture these mobility patterns, thereby bridging the gap between dynamic animal movements and inherently static monitoring infrastructures [49,53,60,68]. In these settings, the integration of remote animal monitoring technologies offers a cost-effective approach to strengthening surveillance capacity, enabling continuous, decentralized data acquisition that reduces dependence on centralized laboratories and supports earlier detection of health anomalies.

Despite these technical possibilities, the low uptake of voluntary control programs in resource-limited territories suggests that economic incentives alone are often insufficient. The cost burden borne directly by farmers often limits participation in optional programs, particularly in areas with fragmented digital infrastructure [26,93]. Within this context, a conceptual framework such as the OH-IS may support risk stratification and more targeted resource allocation; however, its feasibility ultimately depends on political commitment, cross-sectoral governance integration, and sustainable funding mechanisms that extend beyond technological design. Recent institutional analyses further support the economic value of preventive digital health investments, particularly when combined with decentralized and resource-efficient system architectures [36,49]. In this context, enabling continuous, decentralized data acquisition at the farm level reduces dependence on centralized infrastructures and supports earlier detection of health anomalies. This approach is particularly relevant for Inner Areas, where limited access to advanced diagnostics and fragmented digital systems hinder timely surveillance and intervention.

In this context, strengthening local value chains becomes essential, as resilient territorial development in European mountain areas depends on integrated strategies linking agri-food production, environmental stewardship, and socio-economic sustainability [26,28]. Building on this perspective, the integration of federated architectures, lightweight AI models, and sensor-based monitoring systems enables scalable and resource-efficient surveillance solutions across diverse settings. This integrated approach helps reduce disparities between high-resource and resource-limited regions, supporting a more equitable and context-adaptive implementation of the OH paradigm.

### 4.2. Alignment with EU Policy and Global Health Security

The proposed OH-IS architecture is designed to support the regulatory requirements of the EU Animal Health Law [46] and the Farm to Fork Strategy [47], which provide the legal and policy foundation for harmonized animal health governance and sustainable food systems across Member States [24]. These frameworks emphasize the importance of transparency, interoperability, and data-driven decision-making to strengthen preventive approaches, ultimately safeguarding food safety, animal welfare, and cross-border health security [13,32]. By promoting coordinated data governance and cross-sectoral integration, they offer a structural basis to address the regulatory fragmentation and rule misalignment identified in current OH systems [4].

To fully embed this framework within the broader policy landscape, it is essential to align with existing multi-level governance architectures. At the global level, the One Health Joint Plan of Action (2022–2026) of the WHO–FAO–WOAH–UNEP Quadripartite provides the strategic framework for coordinated action across prevention, preparedness, integrated surveillance, governance, and capacity building, spanning human, animal, plant, and environmental health. Complementing this, the recently established Cross-agency One Health Task Force (EEA, ECDC, ECHA, EFSA, and EMA) further strengthens inter-agency coordination for cross-sectoral risk governance under the 2024–2026 action framework. Within this policy landscape, the European Green Deal and the Farm to Fork Strategy should be viewed not as parallel initiatives but as enabling pillars for OH implementation, since sustainability, biodiversity protection, ecosystem restoration, and climate adaptation represent fundamental determinants of health across domains. In this context, the OH-IS can serve as a conceptual bridge, linking environmental status, risk indicators, and agri-food system performance in a spatially explicit, data-driven manner.

A concrete example of this approach is represented by regional brucellosis eradication policies implemented in the Campania Region (southern Italy), where targeted measures, including environmental control, biosecurity protocols, and territory-specific interventions, have been integrated within a One Health framework to address local risk factors [23,60,63].

From an operational standpoint, this vision relies on the consolidation of existing European data and surveillance infrastructures, including EFSA–ECDC zoonoses reporting systems [14], antimicrobial resistance networks, alert mechanisms, and the emerging European Health Data Space (EHDS). The 2025 EHDS regulation [52], in particular, sets a strong regulatory precedent for interoperability, controlled access, and data reuse [94]. By extending these principles to the OH domain, the OH-IS could function as a federated, cross-sectoral data architecture that enhances information exchange across public health, veterinary, and environmental authorities. This structure directly addresses current institutional barriers, facilitating the integration of human, animal, and environmental datasets while supporting a clear shift from reactive surveillance to coordinated, multi-sectoral risk assessment.

Moreover, alignment with the EU AI Act ensures that AI-driven surveillance systems operate within a framework of transparency, accountability, and risk-based regulation. This is essential for ensuring that predictive models are reliable, interpretable, and compliant with European standards for Trustworthy AI, particularly through the implementation of equitable and privacy-preserving architectures [36]. Collectively, these policy frameworks offer a scalable pathway for implementing interoperable surveillance systems across diverse contexts, including Inner Areas, thereby reducing territorial disparities and supporting a more equitable application of the OH paradigm at both regional and global scales. This harmonization is particularly critical for food chain safety, as highlighted in EFSA–ECDC zoonoses reports [14], integrated monitoring across human, animal, food, and feed systems is essential to detect emerging trends, identify transmission sources, and track cross-border pathways. From a climate perspective, this multi-layered integration further supports earlier outbreak detection by revealing the ecological and production drivers that underpin disease emergence and re-emergence.

### 4.3. Ethical Governance and Stakeholder Trust

The implementation of AI in OH surveillance raises key challenges related to data privacy, transparency, and accountability, particularly in systems integrating sensitive human, animal, and environmental data across jurisdictions. To ensure stakeholder trust, the proposed OH-IS would adopt a Human-in-the-Loop governance model, where AI-generated risk signals support, rather than replace, expert decision-making. XAI, as outlined in Section 3, ensures that model outputs are transparent, interpretable, and auditable, mitigating the risks associated with black-box systems.

This approach aligns with international guidelines for ethical AI in health, emphasizing transparency, human oversight, and equity [37]. The use of privacy-preserving architectures such as FL supports decentralized data processing while maintaining data sovereignty, addressing key barriers related to restricted data access in current OH systems. To mitigate bias, the proposed OH-IS would integrate fairness-aware modeling strategies that account for territorial disparities in data availability and digital capacity, thereby reducing the risk of unequal outcomes and ensuring compliance with emerging regulations such as the EU AI Act [95]. Recent studies further highlight that AI systems may introduce biases or misleading interpretations if not properly validated, underscoring the need for transparent, auditable, and reproducible modeling approaches in public health applications [39,40]. For this reason, ethical governance should be considered a core design principle, enabling sustainable adoption and long-term trust in AI-enabled public health systems.

While AI-enabled systems provide powerful tools for integrating heterogeneous data, they cannot replace ground-truth validation, ecological expertise, or the socio-political commitment required for effective data sharing [4]. In complex and dynamic ecological systems, challenges such as data quality limitations (“garbage-in, garbage-out”), algorithmic drift, and surveillance bias, where monitoring prioritizes what is easily digitalized over what is epidemiologically relevant, may compromise the reliability of AI-generated outputs [58]. Additional challenges include governance complexity across jurisdictions, cybersecurity requirements for sensitive health and environmental data, computational constraints in resource-limited settings, and scalability limitations of Federated Learning when data sources are highly heterogeneous. Differences in data quality, metadata completeness, and institutional standards may further affect model performance and interoperability [36,38]. Collectively, these challenges highlight the importance of iterative validation, stakeholder engagement, and continuous monitoring throughout the implementation process. In this context, future implementations should also address uncertainty quantification, false-positive alerts, and model calibration to avoid unnecessary alerts or missed early warning signals. These limitations underscore the need for hybrid approaches that integrate technological innovation with domain expertise and robust governance frameworks.

### 4.4. Toward Scalable and Adaptive Governance

The OH-IS framework is designed as a modular and scalable architecture, enabling phased implementation from pilot territories to broader deployment through FL networks. This adaptive approach supports continuous validation, stakeholder feedback integration, and iterative policy refinement, ensuring responsiveness to evolving epidemiological and environmental conditions [28,30]. By combining federated architectures, FAIR-based data integration, and real-time data acquisition, the proposed OH-IS is intended to address key structural limitations of current OH systems, including data fragmentation, limited interoperability, and restricted accessibility. This facilitates a shift from reactive surveillance to adaptive and anticipatory governance. Systematic assessments confirm that most existing surveillance systems lack integrated data flows, standardized metadata, and real-time feedback mechanisms, constraining their ability to support anticipatory governance [51]. The OH Surveillance Codex further highlights that, without interoperability standards and shared governance frameworks, systems remain sectoral and fragmented, reinforcing the need for coordinated, cross-disciplinary data ecosystems [4,50].

Importantly, the modular nature of the framework allows tailored implementation across heterogeneous territorial contexts, including Inner Areas, where infrastructural constraints and limited resources require flexible and lightweight solutions. In these settings, the integration of AI-driven analytics and decentralized monitoring systems supports scalable deployment without the need for extensive centralized infrastructures [26,28,49]. Recent advances in FAIR-based environmental data ecosystems further highlight the importance of integrating time-series data for dynamic monitoring and predictive modeling of environmental processes [82].

By integrating technological innovation with ethical governance, economic sustainability, and regulatory alignment, the proposed framework offers a concrete pathway to transition OH from a conceptual paradigm toward an operational, data-driven system. Long-term success will depend on reducing institutional fragmentation, harmonizing cross-sectoral data governance, and strengthening collaboration across public health, veterinary, and environmental domains. International frameworks, such as the G20 OH conceptual approaches, highlight the need for coordinated, multisectoral prevention systems capable of integrating human, animal, and environmental data at global scale [2,96]. Recent analyses further emphasize that shifting from reactive outbreak response to predictive surveillance requires sustained investment in interoperable data infrastructures and governance mechanisms [17]. Coordinated, multi-sectoral investment in interoperable OH-IS infrastructures is therefore critical. Only through sustained policy commitment, ethical governance, and technological integration can surveillance systems evolve toward anticipatory, resilience-oriented governance, ultimately safeguarding public health, food security, and ecosystem stability against emerging biological threats.

Finally, the OH scope should extend beyond zoonoses to include plant health, ecosystem disruptions, and agri-food system vulnerabilities, ensuring that predictive prevention translates into resilience, food security, and the preservation of territorial economic value. The climate dimension must be prospectively integrated through scenario-based modeling frameworks [97,98,99]. Representative Concentration Pathways (RCPs), embedded within the SSP–RCP framework of the Intergovernmental Panel on Climate Change (IPCC), provide radiative forcing trajectories that enable the assessment of differentiated risk profiles across health, veterinary, agronomic, and ecosystem domains [99]. This approach allows models to be trained across multiple scenarios and supports ex ante evaluation of adaptation strategies. Under moderate mitigation pathways, systems retain greater adaptive capacity, enabling predictive prevention to focus on localized hotspots and seasonal risks, while containing long-term adaptation costs. Accordingly, the OH-IS should be designed to simulate future vulnerabilities and prioritize interventions across alternative climate trajectories.

## 5. Conclusions and Future Directions

The operationalization of the OH approach is no longer optional but a necessity in an increasingly interconnected world. However, as evidenced by the persistent challenges in controlling endemic diseases such as brucellosis and the delayed responses to multi-country outbreaks such as *Salmonella* Umbilo, current surveillance systems remain constrained by reactive protocols, sectoral silos, and fragmented data governance. In this perspective, transitioning conceptually to an AI-enabled OH-IS represents a critical step toward bridging these operational gaps. This perspective prioritizes interoperable, privacy-preserving, and governance-oriented data integration as a foundation for future AI-enabled One Health surveillance. By conceptually integrating existing methodologies such as FL, FAIR data principles, and XAI, the proposed framework provides a conceptual blueprint for integrating environmental, epidemiological, and territorial drivers into predictive surveillance models. This approach helps address the “digital divide” through lightweight, privacy-preserving technologies while enabling proactive, transparent, and economically sustainable monitoring. The integration of economic sustainability principles and alignment with emerging EU regulatory frameworks, including the Artificial Intelligence Act and the European Health Data Space (EHDS), provides a foundation for moving beyond isolated pilot initiatives. Importantly, the framework supports the revitalization of vulnerable Inner Areas by promoting fairness-aware modeling and decentralized data processing, contributing to overcoming the low uptake of voluntary control programs and reducing territorial disparities in disease surveillance, particularly in high-density livestock systems such as water buffalo farming. However, technology alone is insufficient. Long-term success will depend on reducing institutional fragmentation, strengthening cross-sectoral data governance, and fostering sustained collaboration among public health, veterinary, agricultural, and environmental sectors. Future research should prioritize the empirical validation of OH-IS components through targeted pilot implementations in high-risk territories, including the assessment of cross-sectoral interoperability, governance mechanisms, and predictive performance, alongside the development of standardized cross-border data-sharing agreements and capacity-building initiatives for local health authorities. Coordinated multi-sectoral investment in interoperable OH-IS infrastructures is therefore essential to shift from emergency response to precision-based prevention, where climate change is treated as a structural parameter in risk assessment rather than as a background variable. Consequently, a mature OH-IS framework must evaluate food safety and supply security as co-evolving processes, recognizing that climatic stressors simultaneously amplify biological and chemical hazards while destabilizing territorial value chains. Only through sustained policy commitment, ethical governance, and technological integration can surveillance systems transition from reactive crisis management to anticipatory governance, ultimately safeguarding public health, global food security, and ecosystem resilience against emerging biological threats.

## Figures and Tables

**Table 1:** Comparison between legacy surveillance systems and the proposed AI-enabled OH-IS framework.

Feature	Legacy Surveillance Systems	Proposed AI-Enabled OH-IS Framework
Data Architecture	Centralized, siloed, and fragmented	Federated, interoperable, and decentralized
Environmental Scope	Limited (farm-level proximity)	Extended (water systems, including hydrographic networks and plastisphere dynamics)
Analytical Approach	Descriptive and reactive (post-outbreak)	Predictive and anticipatory (AI-driven risk modeling)
Interoperability	Manual requests and non-standardized metadata	Automated semantic mapping (FAIR principles and OBO ontologies)
Real-Time Capability	Delayed reporting and retrospective analysis	Real-time data integration and continuous monitoring
Governance Model	Top-down, bureaucratic, and opaque systems	Human-in-the-loop with explainable AI (XAI) approaches
Decision-Making Support	Limited, retrospective decision support	AI-driven, predictive, and evidence-based decision support
Economic Logic	Crisis-driven, with high reactive costs	Preventive, based on the “In-Embryo” financial model (ROI-oriented)
Regulatory Alignment	Fragmented national and sectoral rules	Aligned with EU AI Act, EHDS, and Farm to Fork Strategy
Pathogen Focus	Host-centric (animal and human)	Multi-matrix (including environmental carriers and transmission pathways)
Data Privacy	Centralized storage, with higher breach risk	FL (data remain local and GDPR-compliant)
Scalability	Limited to high-resource settings	Scalable through lightweight models for Inner Areas and low- and middle-income countries (LMICs)

Note: Some functionalities reported for the proposed OH-IS framework, including the economic assumptions associated with the “In-Embryo” Financial Model, represent conceptual, non-formalized, and anticipated capabilities derived from the architecture described in this manuscript and require future empirical validation through pilot implementations, performance assessments, and real-world deployment studies.

## Data Availability

The data and examples reported in the manuscript are derived from the authors’ previously published studies, recent peer-reviewed literature, and publicly available institutional sources cited throughout the manuscript. These include EFSA–ECDC reports, EU regulatory repositories such as EUR-Lex, WHO/FAO/WOAH/UNEP One Health policy documents, and EFSA/EEA climate and environmental risk assessments. No unpublished external datasets, proprietary databases, or additional data platforms were used for original data analysis in this Perspective Article. All supporting sources are cited in the reference list.
